# Efficient Virus-Induced Gene Silencing (VIGS) Method for Discovery of Resistance Genes in Soybean

**DOI:** 10.3390/plants14101547

**Published:** 2025-05-21

**Authors:** Kelin Deng, Zihua Lu, Hongli Yang, Shuilian Chen, Chao Li, Dong Cao, Hongwei Wang, Qingnan Hao, Haifeng Chen, Zhihui Shan

**Affiliations:** 1 Oil Crops Research Institute, Chinese Academy of Agricultural Sciences, Wuhan 430062, China; 18369696664@163.com (K.D.); 13604092003@163.com (Z.L.); yanghongli@caas.cn (H.Y.); chenshuilianjy@163.com (S.C.); lichao06@caas.cn (C.L.); caodong@caas.cn (D.C.); shanzhihui@caas.cn (Z.S.); 2 Graduate School, Chinese Academy of Agricultural Sciences, Beijing 100081, China; 3 Key Laboratory of Biology and Genetics Improvement of Oil Crops, Ministry of Agriculture and Rural Affairs, Wuhan 430062, China; 4 Department of Plant Genetics and Breeding, College of Agronomy, Shandong Agricultural University, Taian 271018, China; wanghongwei@sdau.edu.cn

**Keywords:** soybean, TRV–VIGS, *GmPDS*, gene screening, functional validation

## Abstract

Soybean (*Glycine max* L.) is a vital grain and oil crop, serving as a primary source of edible oil, plant-based protein, and livestock feed. Its production is crucial for ensuring global food security. However, soybean yields are severely impacted by various diseases, and the development of disease-resistant cultivars remains the most sustainable strategy for mitigating these losses. While stable genetic transformation is a common approach for studying gene function, virus-induced gene silencing (VIGS) offers a rapid and powerful alternative for functional genomics, enabling efficient screening of candidate genes. Nevertheless, the application of VIGS in soybean has been relatively limited. In this study, we established a tobacco rattle virus (TRV)-based VIGS system for soybean, utilizing *Agrobacterium tumefaciens*-mediated infection. The TRV vector was delivered through cotyledon nodes, facilitating systemic spread and effective silencing of endogenous genes. Our results demonstrate that this TRV–VIGS system efficiently silences target genes in soybean, inducing significant phenotypic changes with a silencing efficiency ranging from 65% to 95%. Key genes, including phytoene desaturase (*GmPDS*), the rust resistance gene *GmRpp6907*, and the defense-related gene *GmRPT4*, were successfully silenced, confirming the system’s robustness. This work establishes a highly efficient TRV–VIGS platform for rapid gene function validation in soybean, providing a valuable tool for future genetic and disease resistance research.

## 1. Introduction

Soybean seeds are rich in oil and protein, making them a valuable resource for food production, animal feed, and industrial applications. To meet the growing global demand, agricultural advancements have focused on developing high-yielding cultivars, optimizing cultivation practices, and expanding arable land for soybean production [1]. However, soybean plants frequently encounter various environmental stressors during their growth cycle, with pest infestations and disease outbreaks posing particularly significant challenges to crop productivity [2]. These factors represent major constraints that significantly limit soybean growth and development, while also severely compromising both yield and quality. Consequently, enhancing soybean resilience against various stress factors has emerged as a critical challenge in contemporary breeding programs [3]. Cultivating high-quality soybean germplasm with enhanced disease and stress resistance has emerged as the most economical and effective strategy. Consequently, identifying and characterizing key genes associated with these traits is crucial for developing novel soybean germplasm through molecular breeding approaches.

The most effective way to identify gene function is to look at the phenotypic variation that occurs at the cellular and global level when gene expression is blocked or enhanced. The analysis of soybean gene function usually adopts the method of genetic transformation to obtain the overexpressed or silenced (RNAi) mutant of the target gene, and obtaining the overexpressed or functionally deficient mutant of the target gene is a very effective way to study gene function. However, the genetic transformation of soybean is very time-consuming and laborious work. Therefore, the establishment of a safe, efficient, and stable analysis system for soybean gene function is best for the study of soybean gene function and the cultivation of excellent soybean seed quality. VIGS is a form of post-transcriptional gene silencing (PTGS), which has become a very useful means for people to analyze the function of genes more quickly and accurately and analyze the intricate links and interactions between genes [4,5,6,7].

VIGS has emerged as an effective tool for functional genomic studies in soybean, particularly to circumvent the challenges associated with stable genetic transformation. Several viral vectors have been developed for VIGS in soybean, including those derived from pea early browning virus (PEBV) [8], soybean yellow common mosaic virus (SYCMV) [9], bean pod mottle virus (BPMV) [10], apple latent spherical virus (ALSV) [11], and cucumber mosaic virus (CMV) [12]. Among these, the BPMV-based silencing system is the most widely adopted due to its efficiency and reliability in soybean. In 2013, Kandoth et al. utilized the bean pod mottle virus (BPMV)-mediated VIGS system to investigate soybean cyst nematode parasitism in *Glycine max* [13]. Subsequently, Cooper et al. demonstrated that BPMV-induced silencing of Rpp1 compromised soybean rust immunity, highlighting the utility of this approach in functional genomics [14]. The BPMV–VIGS system has been employed to identify the *Rsc1-DR* gene, which confers resistance to the soybean mosaic virus strain SC1 (SMV-SC1) [15]. Additionally, silencing *GmBIR1* via BPMV enhanced soybean resistance to SMV, resulting in phenotypes indicative of a constitutively activated defense responses [16]. Furthermore, this system has been employed to validate the role of *Rbs1* in conferring resistance to brown stem rot (BSR) in soybean and to elucidate the oligogenic inheritance underlying this disease [17]. However, the implementation of BPMV–VIGS technology faces substantial technical hurdles, particularly its frequent reliance on particle bombardment. This approach often induces leaf phenotypic alterations, which can interfere with accurate phenotypic evaluation in subsequent analyses.

In contrast, TRV has recently emerged as the most widely adopted viral vector system [18,19]. TRV vectors have been widely utilized in a variety of plant species, including tomato (*Solanum lycopersicum*) [20], tobacco (*Nicotiana* spp.) [21], petunia (*Petunia hybrida*) [22], chili pepper (*Capsicum* spp.) [23], *Arabidopsis thaliana* [24], and cotton (*Gossypium* spp.) [25]. The TRV–VIGS system has elucidated the significant role of tobacco *NtTIFYs* in combating bacterial wilt [26]. The *SlMsrB5* gene in tomato has been implicated in the methyl jasmonate-induced cold tolerance of post-harvest fruits [27]. The *CaWRKY3* gene in pepper has been shown to enhance the immune response to Ralstonia solanacearum by modulating various *WRKY* transcription factors [28]. Additionally, TRV elicited fewer symptoms compared to other viruses, thereby minimizing harm to the plants and preventing the masking of the silencing phenotype [29]. Despite its widespread application in solanaceous crops, reports on the use of TRV-mediated VIGS for functional gene studies in soybean remain limited.

In this study, soybean was employed as a model plant to validate an optimized TRV-mediated VIGS protocol. The modified method utilizes *Agrobacterium tumefaciens*-mediated infection through the cotyledon node, with viral transmission initiating from the cotyledon node to achieve systemic silencing of endogenous genes throughout the plant. This research establishes a robust platform for rapid and efficient functional characterization of critical soybean genes. The findings will contribute to expanding the genetic resources for soybean disease resistance and stress tolerance, ultimately facilitating the development of novel soybean cultivars with enhanced resistance traits.

## 2. Results

### 2.1. Construction of TRV–VIGS Vector

**Figure 1:**
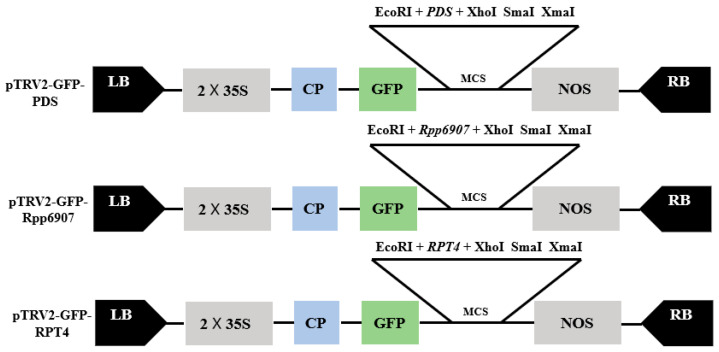
Construction of TRV–VIGS Vector. RdRp, RNA-dependent RNA polymerase; LB and RB, left and right borders of T-DNA; 35S, duplicated CaMV 35S promoter; CP, coat protein; MCS, Multiple cloning sites; GFP, green fluorescent protein; NOS, nopaline synthase terminator.

Using cDNA synthesized from healthy soybean leaves as the template, PCR amplification was performed with the corresponding primers (Table 1). The amplification products were analyzed by electrophoresis, which revealed distinct bands of the target gene. The PCR-amplified target fragment was then ligated into the pTRV2–GFP vector, which had been digested with *EcoRI* and *XhoI* restriction enzymes. The ligation product was transformed into DH5α competent cells, and positive clones were selected for sequencing. Recombinant plasmids with confirmed correct sequences were extracted and subsequently introduced into *Agrobacterium tumefaciens* GV3101. A schematic diagram of the constructed recombinant vector is shown in Figure 1.

### 2.2. Agroinfiltration Methods

**Figure 2:**
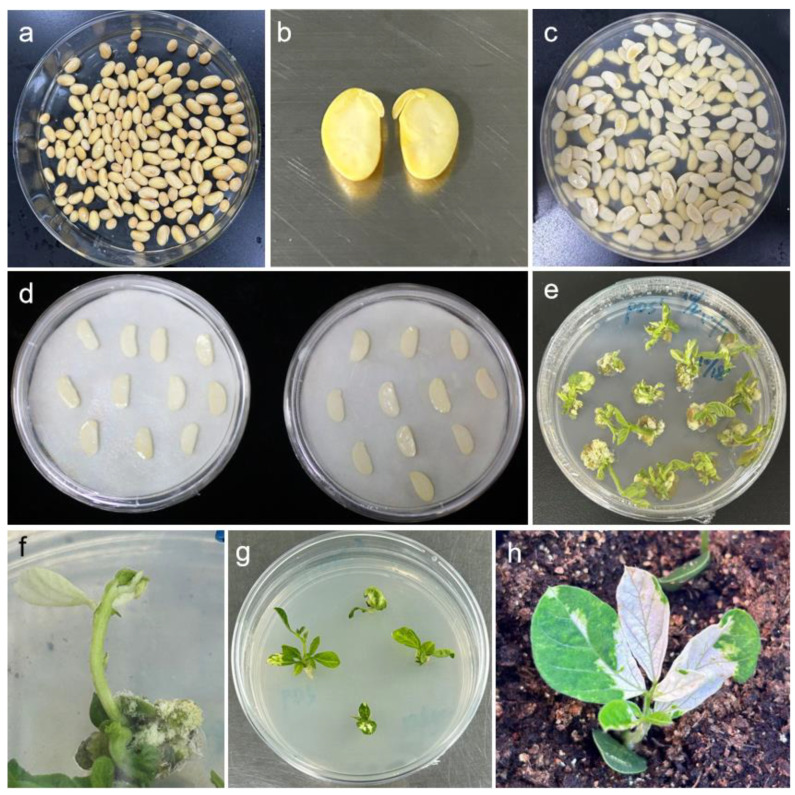
TRV–VIGS infection process. (**a**) Seed imbibition for 5–6 h; (**b**) preparation of half-seed explants; (**c**) soaking of half-seed explants in *Agrobacterium* suspension; (**d**) co-cultivation in darkness; (**e**) transfer to induction medium; (**f**) selection of suitable seedlings; (**g**) seedling growth and medium replacement; (**h**) seedling transplantation.

Conventional methods (misting and direct injection) showed low infection efficiency due to soybean leaves’ thick cuticle and dense trichomes, as they impeded liquid penetration. Our optimized protocol involved soaking sterilized soybeans in sterile water until swollen, longitudinally bisecting them to obtain half-seed explants, then infecting fresh explants by immersion for 20–30 min (optimal duration) in Agrobacterium tumefaciens GV3101 suspensions containing either pTRV1 or pTRV2–GFP derivatives (pTRV:empty [pTRV1 + pTRV2–GFP], pTRV:GmPDS [pTRV1 + pTRV2–GFP-GmPDS], pTRV:Rpp6907 [pTRV1 + pTRV2–GFP-Rpp6907], or pTRV:RPT4 [pTRV1 + pTRV2–GFP-RPT4]). The sterile tissue culture-based procedure (Figure 2) achieved transformation efficiencies evaluated by qPCR-detected GFP expression, with samples exceeding reference thresholds scored as positive.

### 2.3. GFP Fluorescence Evaluation of Agrobacterium Infection Efficiency

**Figure 3:**
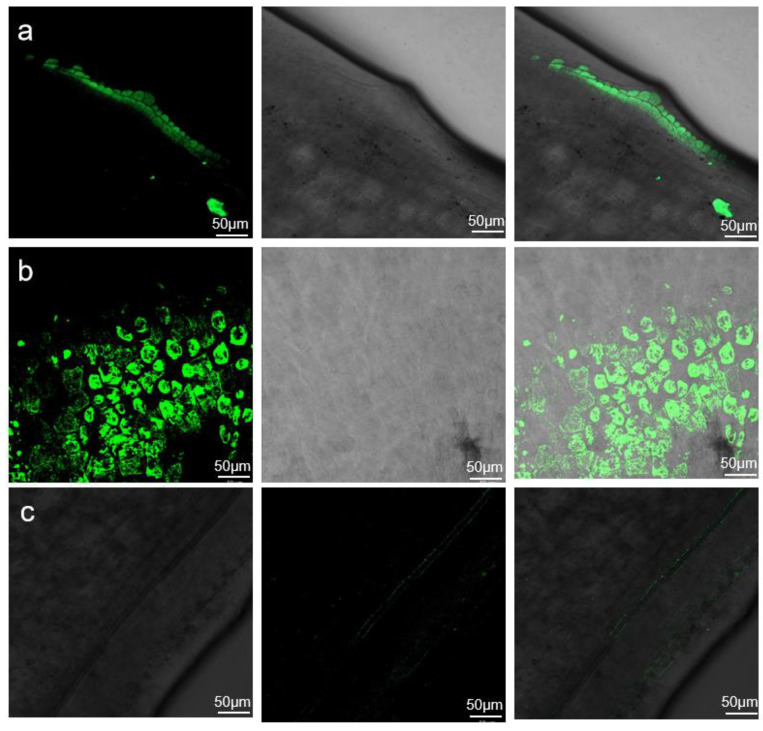
Fluorescence detection of viral infection. (**a**) Longitudinal section at 4 days post-infection (dpi), showing initial infection in 2–3 cell layers; (**b**) transverse section at 4 dpi, demonstrating widespread infection across most cells; (**c**) negative control for airborne transmission, with no detectable fluorescence signal.

To investigate the effects of *Agrobacterium* infection on the cotyledonary node, a portion of the hypocotyl from each explant was excised under sterile conditions on the fourth day post-infection and observed under a fluorescence microscope. The results showed fluorescence signals at the sites of successful infection (Figure 3). The longitudinal section revealed that the infection initially infiltrated 2–3 cell layers before gradually spreading to deeper cells. In the transverse section, more than 80% of the cells exhibited successful infiltration, indicating a high infection efficiency (Figure 3a,b). In contrast, plants that failed to be infected showed no detectable fluorescence (Figure 3c). Using this method, the effective infectivity efficiency exceeded 80%, reaching up to 95% for Tianlong 1.

### 2.4. GmPDS Silencing in Soybean

**Figure 4:**
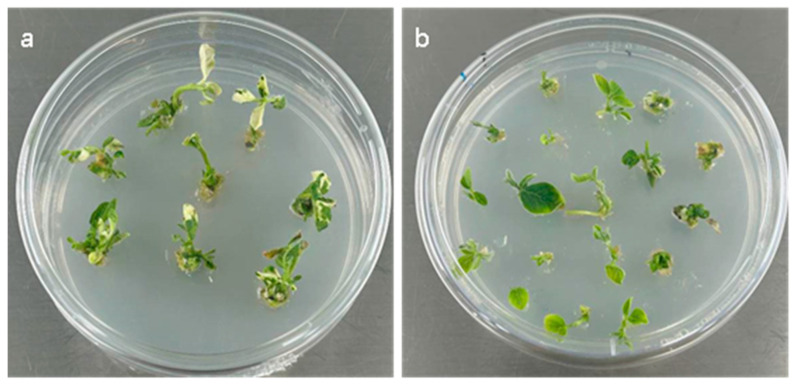
Phenotypic changes in Tianlong 1 plants after *GmPDS* gene silencing. (**a**) Tianlong 1 seedlings infected with the pTRV:GmPDS recombinant vector exhibited leaf whitening and phenotypic alterations at 21 days post-infection.; (**b**) control seedlings infected with the empty TRV vector showed neither leaf whitening nor phenotypic changes after 21 days.

To evaluate the overall effectiveness of the optimized TRV-based VIGS protocol for gene silencing in the soybean cultivar Tianlong 1, we assessed the silencing efficiency of *GmPDS* through phenotypic observation and expression analysis. Following treatment, photobleaching was observed in leaves inoculated with pTRV:*GmPDS* at 21 days post-inoculation (dpi), while no such phenotype was detected in pTRV:*empty* controls (Figure 4a,b). The photobleaching phenotype initially appeared in the cluster buds. Seedlings from the pTRV:*GmPDS* treatment group were subsequently transplanted into nutrient soil for further growth. Notably, some seedlings that initially showed no photobleaching gradually developed the phenotype after two weeks, potentially due to delayed viral particle transport within vascular bundles. Quantitative analysis revealed a photobleaching efficiency of 53.3–73.3% for *GmPDS* silencing (Table S1). Additionally, qPCR analysis confirmed significant downregulation of *GmPDS* gene expression.

**Figure 5:**
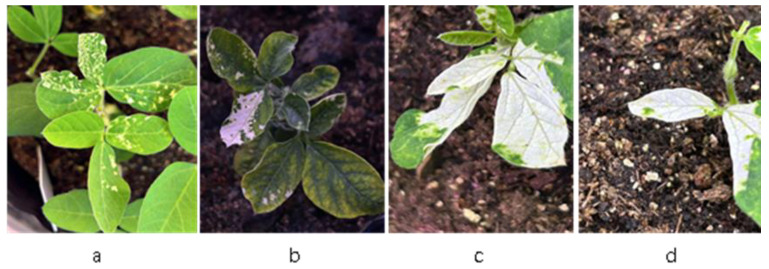
Phenotypic alterations in PDS-silenced adult soybean plants after transplantation. (**a**,**b**) Mottled chlorotic symptoms observed on soybean leaves; (**c**,**d**) complete leaf whitening phenotype in soybean plants.

Successful silencing of *GmPDS* via TRV–VIGS generated a spectrum of photobleaching phenotypes, ranging from greenish-white mosaic patterns to complete albinism (Figure 5). To quantitatively assess silencing efficiency and its phenotypic effects, we performed qPCR analysis on three phenotypically defined groups within the same experimental population: (i) fully albino plants, (ii) chlorotic plants, and (iii) empty vector controls.

**Figure 6:**
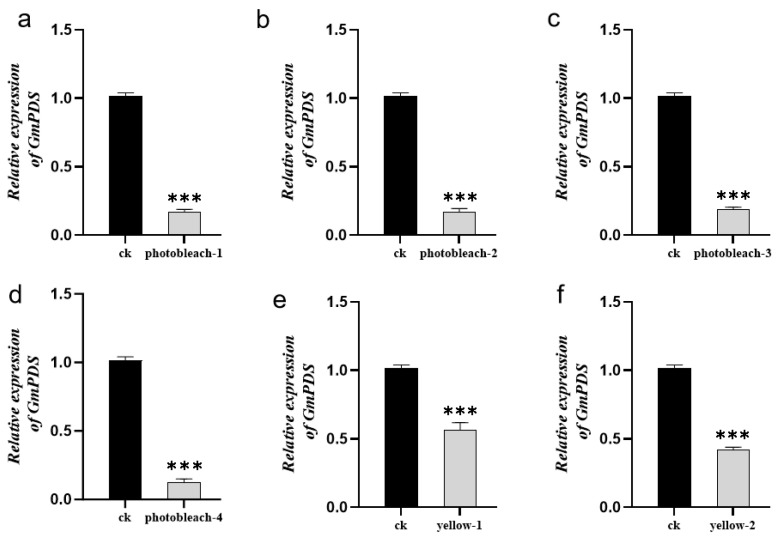
Expression levels of *GmPDS* genes in plants with different phenotypes. (**a**–**d**) Relative expression levels of *GmPDS* in leaves of photobleaching plants (No. 1–4); (**e**,**f**) relative expression levels of GmPDS in leaves of chlorotic yellowing plants (No. 1–2). For this experiment, seven Tianlong 1 leaves exhibiting the respective phenotypes were pooled as one biological replicate, with three independent biological replicates performed. CK: pTRV-empty. Error bars represent the mean ± SE of independent biological replicates. All statistical analyses were performed with GraphPad Prism (v.9.5). A Student’s *t*-test was used to assess differences between different treatments (*** *p* < 0.001).

The analysis revealed a strong dose–response relationship between *GmPDS* transcript levels and phenotypic severity. Albino plants showed the most pronounced gene suppression (83.7% ± 2.9% reduction versus controls), while chlorotic plants exhibited intermediate silencing (52.5% ± 8.0% reduction) (Figure 6). Statistical analysis confirmed a significant positive correlation between the degree of gene silencing and phenotypic intensity, demonstrating that complete albinism requires near-complete (>80%) transcript depletion whereas partial chlorosis occurs at moderate (50–60%) silencing levels.

### 2.5. Systematic Silencing of TRV–VIGS

**Figure 7:**
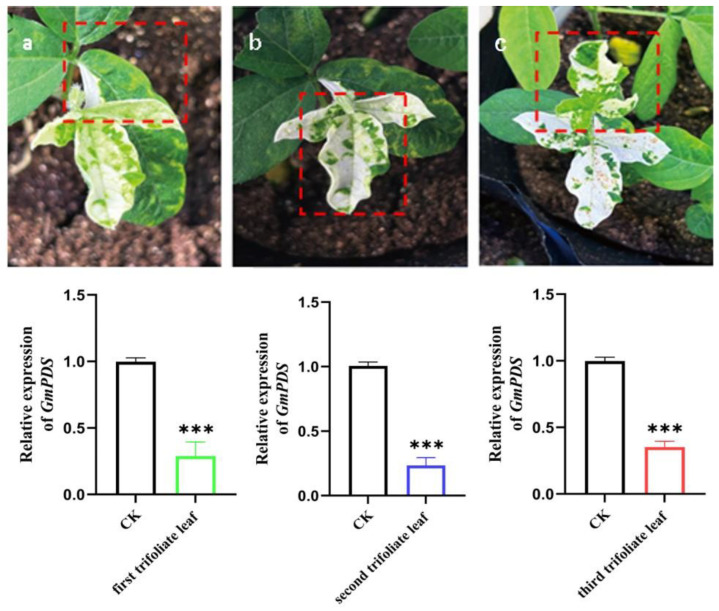
Phenotypic changes in Tianlong 1 plants following *GmPDS* gene silencing. (**a**) Phenotype and silencing efficiency of *GmPDS* in the first trifoliate leaf, showing photobleaching; (**b**) phenotype and silencing efficiency of *GmPDS* in the second trifoliate leaf, showing photobleaching; (**c**) phenotype and silencing efficiency of *GmPDS* in the third trifoliate leaf, showing photobleaching. The red box highlights trifoliate leaves at different nodes. The experiment set-up included three independent biological replicates. The error bar is the mean ± SE of independent biological replicates. All statistical analyses were performed with GraphPad Prism (v.9.5). A Student’s *t*-test was used to assess differences between different treatments (*** *p* < 0.001).

To evaluate the systemic gene silencing efficacy of the TRV–VIGS system in soybean plants, we performed dynamic multi-leaf sampling and quantitative gene expression analysis on plants displaying photobleaching phenotypes. As illustrated in Figure 7, both phenotypic progression and *GmPDS* gene expression levels were monitored across the first to third trifoliate leaves.

Upon successful viral infection and the onset of whitening symptoms in the first trifoliate leaf, subsequent photobleaching development in newly emerged second and third trifoliate leaves confirmed the systemic mobility of the TRV–VIGS system following initial infection.

qPCR analysis of leaves at different nodal positions demonstrated significantly reduced *GmPDS* expression levels in photobleached leaves (first to third trifoliate) compared with control plants. The photobleaching phenotype consistently co-occurred with leaf curling, a hallmark symptom of viral infection in plants. Notably, the stems exhibited no visible photobleaching.

### 2.6. Application of TRV–VIGS System

#### 2.6.1. Functional Analysis of the GmRpp6907 Gene Using the TRV–VIGS System

*GmRpp6907* is a soybean rust resistance gene. In this study, we attempted to silence *GmRpp6907* in the cultivar SX6907. To evaluate the silencing efficiency at the molecular level, the expression of *GmRpp6907* was analyzed by qPCR.

**Figure 8:**
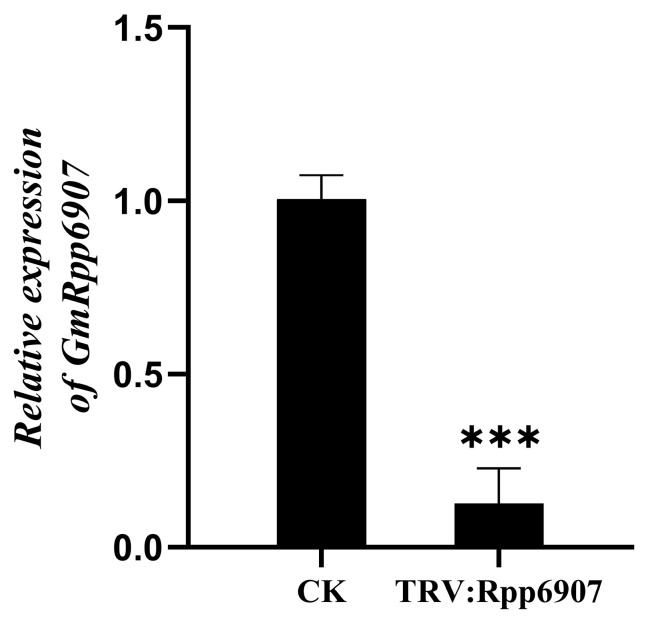
An expression analysis following *GmRpp6907* gene silencing. Ten plants were selected as one biological replicate in this experiment, and the three independent biological replicates were set. Error bars represent the mean ± SE of independent biological replicates. All statistical analyses were performed with GraphPad Prism (v.9.5). A Student’s *t*-test was used to assess differences between different treatments (*** *p* < 0.001).

At 24 days post-infection with either pTRV:*GmRpp6907* or the empty vector control (pTRV:empty, CK), a significant difference in *GmRpp6907* expression levels was observed. The results demonstrated that *GmRpp6907* expression in pTRV:*GmRpp6907*-infected plants was reduced by 87.31% ± 10.1% compared to the control (Figure 8).

**Figure 9:**
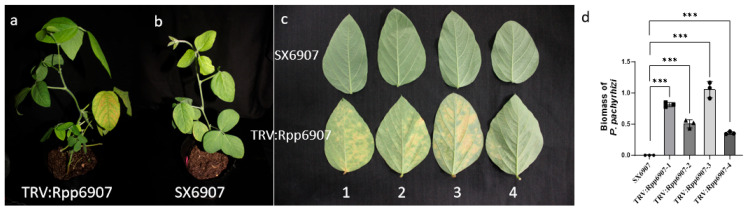
Phenotypic characteristics of SX6907 following GmRpp6907 gene silencing. (**a**) Growth performance of SX6907 plants post-*GmRpp6907* gene suppression; (**b**) control phenotype of SX6907 plants infected with TRV:empty (**c**) comparative analysis of *P. pachyrhizi* susceptibility in GmRpp6907-silenced vs. control SX6907 leaves; (**d**) biomass analysis of *P. pachyrhizi* in infected soybean using qPCR. Infected leaves were collected 14 d (*P. pachyrhizi*) after inoculation and used for RNA isolation. Colonization of *P. pachyrhizi* was quantified by qPCR and normalized to the soybean ubiquitin gene. Asterisks indicate significant differences based on Student’s *t*-test (*** *p* < 0.001). Experiments were repeated three times with similar results.

Leaves with silenced *GmRpp6907* were inoculated with the *P. pachyrhizi* isolate SS4. At 7–10 days post-inoculation (dpi), the *GmRpp6907*-silenced plants exhibited a shift from an immune phenotype to a susceptible phenotype, displaying varying degrees of disease severity. The susceptibility level was positively correlated with the reduction in *GmRpp6907* expression. In contrast, control plants retained a rust-resistant phenotype with no rust spore production observed (Figure 9c). In line with this, the *P. pachyrhizi* biomass was significantly increased in the samples after the *GmRpp6907* gene was silenced compared with SX6907 (Figure 9d). These results confirmed that the *GmRpp6907* gene was successfully silenced.

#### 2.6.2. Functional Identification of GmRPT4

*GmRPT4* serves as a pivotal regulatory subunit of the 26S proteasome, tasked with the recognition and degradation of ubiquitinated proteins associated with defense mechanisms, thus playing a crucial role in the dynamic regulation of plant immune signaling homeostasis. Through its mediation of the selective degradation of pathogen effector proteins, RPT4 facilitates the activation of the Effector-Triggered Immunity (ETI) pathway and augments the hypersensitive response (HR), thereby limiting pathogen proliferation and contributing significantly to plant stress resistance.

**Figure 10:**
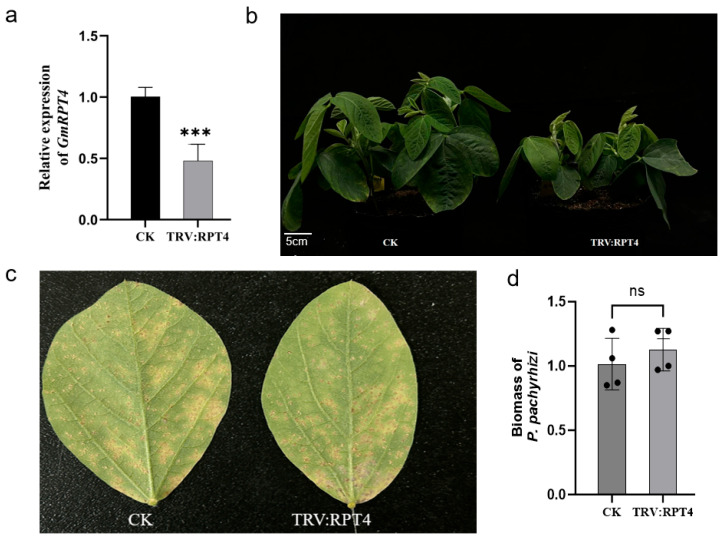
Comparative analysis of Tianlong 1 plants before and after *GmRPT4* silencing. (**a**) Expression analysis of *GmRPT4* after gene silencing (*n* = 7); (**b**) growth performance of SX6907 plants following *GmRPT4* suppression; (**c**) comparative analysis of *P. pachyrhizi* susceptibility in GmRpp6907-silenced vs. control SX6907 leaves; (**d**) biomass analysis of *P. pachyrhizi* in infected soybean using qPCR. Infected leaves were collected 14 d after inoculation (by *P. pachyrhizi*) and used for RNA isolation. Colonization of *P. pachyrhizi* was quantified by qPCR and normalized to the soybean ubiquitin gene. *** *p* < 0.001, ns indicates no significant differences based on Student’s *t*-test. Experiments were repeated three times with similar results.

The VIGS-mediated silencing of *GmRPT4* in Tianlong 1 resulted in a significant reduction in transcript levels (53.8% ± 9.7%) compared to pTRV–GFP controls at 24 dpi (Figure 10a). Phenotypic analysis revealed that GmRPT4 silencing markedly impaired plant growth and development, leading to a pronounced dwarf phenotype relative to wild-type plants (Figure 10b).

To assess the role of *GmRPT4* in soybean rust resistance, GmRPT4-silenced and control plants were inoculated with SS4 at 25 days post-VIGS. Disease progression was monitored, and silenced plants displayed extensive rust pustules and elevated spore production, phenotypically mirroring the susceptible control (unmodified Tianlong 1) (Figure 10c). qPCR was employed to compare *P. pachyrhizi* biomass accumulation between *GmRPT4*-silenced plants and control groups. Consistent with the phenotypic observations, fungal biomass quantification confirmed no significant difference between silenced plants and susceptible controls, further supporting the lack of enhanced resistance (Figure 10d).

These results indicate that while *GmRPT4* plays a role in regulating soybean growth, it does not contribute to the basal resistance against *P. pachyrhizi*. The unchanged rust susceptibility following *GmRPT4* silencing implies that this gene is not a critical factor in soybean–pathogen interactions, at least within the Tianlong 1 genetic background. Further research is necessary to investigate the role of *GmRPT4* in other stress responses or developmental pathways.

## 3. Discussion

VIGS technology represents a convenient and efficient reverse genetics approach. Unlike RNAi, it eliminates the need for time-consuming and labor-intensive genetic transformation procedures when analyzing gene function. To date, the TRV vector has been extensively utilized for functional gene validation across diverse plant species. Compared to other viral vectors, the TRV–VIGS system demonstrates superior advantages in multiple aspects: longer insert capacity for target genes, higher gene silencing efficiency, prolonged silencing persistence, and minimal adverse effects on host plants [30,31]. Traditionally, VIGS inoculation has been performed through stem infiltration [32], leaf injection [33], or direct application of in vitro transcribed viral RNA onto leaves [34]. In this study, we developed an innovative *Agrobacterium*-mediated infection protocol for soybean cotyledonary node explants. Unlike conventional injection methods, our cotyledon node approach effectively overcomes infection barriers posed by soybean trichomes and dense tissue structures, thereby substantially improving infection efficiency and reducing the time required for VIGS implementation in soybean. This novel method additionally offers excellent systemic transmission, lower operational costs, and reduced leaf damage. The optimized TRV vector serves as a powerful tool for rapid and effective functional characterization of soybean genes, representing a valuable addition to the toolkit for soybean genomics research.

The VIGS system in our laboratory mainly uses a virus to infect the shoot apical meristem (SAM) of plants, to achieve the goal of infecting the entire plant seedling. Therefore, we can appropriately use some methods, such as using specific protein complexes, to enhance transformation efficiency. A previous study utilized the GRF–GIF complex to enhance the transformation efficiency of CRISPR/Cas9, which may be due to the ability of the GRF–GIF complex to increase the size of the SAM and promote stem regeneration during organogenesis [35]. Based on this discovery, we can take advantage of this protein complex to increase the growth of callus and thereby promote the efficacy of VIGS infection.

In this study, we engineered a modified TRV–GFP vector by inserting the GFP coding sequence downstream of the coat protein gene in the TRV2 backbone. This modification enables convenient tracking of viral infection and spread in host plants using a handheld UV light, significantly improving experimental monitoring efficiency. Previous studies have systematically evaluated the gene-silencing efficiency of the TRV–GFP vector across multiple plant species, including *Nicotiana benthamiana*, *Arabidopsis thaliana*, rose, strawberry, and chrysanthemum. Comparative analyses confirmed that the TRV–GFP vector maintains infection efficiency comparable to the original TRV vector in all tested species, indicating that GFP insertion does not impair viral replication or systemic movement [36]. The silencing efficiency of the TRV–GFP vector has also been validated in cassava. Phenotypic and molecular detection results demonstrate the feasibility of this approach, facilitating rapid analysis of cassava functional genomics [37]. Additionally, related studies in gladiolus have shown that the vector effectively induces gene silencing [38].

Thus, the TRV–GFP vector serves as a versatile and efficient VIGS tool, combining the reliability of the original TRV system with the advantage of visual tracking. Its successful applications in cassava and gladiolus highlight its potential to accelerate gene function studies across diverse plant species, advancing crop improvement and molecular plant biology research. In 2022, Aragonés et al. developed an efficient gene silencing and genome editing system using two mini-T-DNA vectors (pLX-TRV1 and pLX-TRV2), which simplifies cloning procedures and supports protein expression and gene silencing even at low bacterial densities [39]. This system has been successfully applied in Nicotiana benthamiana, and we plan to further optimize it for use in soybean to improve gene silencing efficiency.

In this study, we employed the TRV–VIGS system to silence the *GmRpp6907* gene in the rust-resistant soybean germplasm SX6907. This intervention effectively abolished its inherent rust immunity, rendering the plants susceptible to rust disease. The observed susceptibility was attributed to the downregulation of *GmRpp6907* expression following TRV–VIGS infection, which impaired the gene’s ability to activate immune responses against pathogen effector proteins. Furthermore, by combining these findings with the rust resistance observed in transgenic Tianlong 1 soybean plants overexpressing *GmRpp6907* (previously generated in our laboratory), we preliminarily confirmed the disease-resistant function of *GmRpp6907*. Additionally, we silenced the GmRPT4 gene in Tianlong 1 using the TRV–VIGS system, which led to a significant reduction in its expression levels. Although this silencing did not confer resistance to *P. pachyrhizi* (the causal agent of soybean rust), it did negatively impact plant growth. Collectively, this study demonstrates that the TRV–VIGS system provides a straightforward and effective tool for rapid gene screening and functional characterization in plants.

Taking *GmRpp6907* as an example, its rust-resistant phenotype has not only been experimentally confirmed in soybean but also offers critical insights for studying disease resistance genes in other species. Future studies can utilize the TRV–VIGS system to silence candidate resistance genes in soybean. On one hand, this allows for the validation of gene function in soybean disease resistance by comparing phenotypic changes with controls; on the other hand, it can effectively avoid programmed cell death caused by plant “hypersensitivity reactions” due to the overexpression of certain disease-resistance-related genes [40]. The soybean TRV–VIGS system established in this study expands possibilities for screening and breeding rust-resistant soybean germplasm.

With rapid advancements in biotechnology, novel genes with agronomic value are continuously being identified and applied to crop improvement. However, many functional genes remain uncharacterized due to limited validation tools. We anticipate that the soybean TRV–VIGS system will increasingly facilitate rapid screening of elite genes, thereby enhancing its potential applications in soybean quality improvement.

## 4. Materials and Methods

### 4.1. Plant Materials and Soybean Rust Pathogen

Tianlong 1 and SX6907 soybean plants were used in the experiments. The seeds of Tianlong 1 and SX6907 were harvested at the Wuchang Experimental Base in Wuhan, Hubei Province. Tianlong 1 is a soybean variety susceptible to *P. pachyrhizi*. SX6907 is a Chinese landrace with a high resistance to *P. pachyrhizi*. The rust fungus used in the experiment was derived from the virulent strains of *P. pachyrhizi* isolate SS4, which were maintained on soybean Tianlong 1 plants.

### 4.2. Developing TRV–VIGS Constructs

pTRV1 and pTRV2–GFP VIGS vectors were constructed following a previously reported protocol [41]. The pTRV2–GFP vector was based on the construct developed by Tian et al. [36]. All oligonucleotide primers used in this study were synthesized by Sangon Biotech (Shanghai, China), and their sequences are listed in Table 1.

Glycine max phytoene desaturase (*GmPDS*), a key enzyme in the carotenoid biosynthesis pathway, was selected as a visual marker for VIGS, as silencing this gene induces visible chlorosis in plant leaves. For the construction of pTRV2-GFP-PDS, a 301 bp fragment of soybean *PDS* (GenBank: XM_028355994) was amplified from soybean cDNA. Additionally, we targeted two soybean resistance-related genes. The first, *GmRpp6907*, a rust resistance gene cloned from the soybean cultivar SX6907. A 265 bp fragment of *GmRpp6907* was amplified for the construction of pTRV2-GFP-Rpp6907. The second, *GmRPT4* (Glyma.18G036400), is a component of the 26S proteasome regulatory complex. A 301 bp fragment of *GmRPT4* derived from the soybean cultivar Tianlong1 was amplified for pTRV2-GFP-RPT4.

The PCR products were cloned into *EcoR*I-*Xho*I-digested pTRV2-GFP using In-Fusion Cloning technology (Figure 1). The recombinant vectors (pTRV2-GFP derivatives and pTRV1) were then transformed into *Agrobacterium* strain GV3101. Our results demonstrate that we successfully constructed two VIGS vectors for soybean.

### 4.3. Preparation of Infection Solution

For the VIGS experiments, *Agrobacterium* strain GV3101, harboring either pTRV1 or pTRV2-GFP and its derivatives, was used. After transformation, the *Agrobacterium* cells were inoculated onto Luria–Bertani (LB) agar plates supplemented with 100 μg/mL kanamycin (Kan) and 50 μg/mL rifampicin (Rif), followed by incubation at 28 °C for 36–72 h. A 5 mL starter culture was grown overnight at 28 °C in LB medium containing the same antibiotics (100 μg/mL Kan and 50 μg/mL Rif). Then the culture was inoculated into a 50 mL LB medium, containing kanamycin. The cultures were grown overnight on a shaker at 28 °C and 180 rpm. *Agrobacterium* cells were centrifuged at 5500× *g* for 10 min and *Agrobacterium* cells were harvested and resuspended into infiltration medium (200 μmol/L acetosyringone, 0.1 mol/L MgCl_2_), adjusted to an OD600 of 0.6. Finally, pTRV1 and pTRV2-GFP (or its derivatives) were mixed in a 1:1 ratio, stabilized at room temperature for 3 h, and shaken well before use.

### 4.4. Explant Preparation and Infection

Soybean seeds were surface-sterilized by incubation in a 150 mm Petri dish containing a mixture of 4 mL HCl and 100 mL NaClO for 8 h. After sterilization, the seeds were transferred to a sterile 90 mm Petri dish, supplemented with an appropriate amount of sterile water, and incubated at 28 °C for 4–5 days to promote germination. To prepare explants, a longitudinal incision was made along the hilum under sterile conditions, and the seed coat was carefully removed. The embryonic axis was then excised at the 3–5 mm junction between the hypocotyl and cotyledon to obtain half-seed explants. These explants were immersed in *Agrobacterium* inoculum for 20–30 min and subsequently transferred to a 150 mm Petri dish lined with filter paper moistened with 5 mL of MES infiltration solution.

### 4.5. Explant Co-Cultivation

The cotyledons of the soybean explants were placed flat-side down in close contact with the filter paper, with 20 explants arranged per dish at uniform spacing. The Petri dishes were sealed airtight and incubated in complete darkness at 23 °C for 3–5 days. Following co-cultivation, the explants were transferred to fresh rooting medium to promote further development. Once adventitious roots had elongated, the plantlets were acclimatized and transplanted into nutrient-rich soil under controlled growth conditions (14-h light/10-h dark photoperiod, 70% relative humidity, and 12,000 lux light intensity).

### 4.6. Fluorescence Observation

The explants were inoculated with the TRV–VIGS system harboring a GFP tag. The experiment included three biological replicates, with 30 explants per replicate. Post-inoculation, fluorescence in the explants was monitored and documented daily.

Initial fluorescence screening was performed using a LUYOR-3415RG (Luyang Biotechnology Co., Ltd., Shanghai, China) handheld GFP excitation light source to detect GFP signals in infected explants. This device was used to observe fluorescence in hypocotyls, adventitious roots, and mature leaves at later growth stages.

For detailed fluorescence analysis, tissue sections were prepared and examined under an OLYMPUS upright fluorescence microscope BX53 (Olympus Corporation, Nagano, Japan). Briefly, hypocotyl or cotyledon samples (~0.5 cm) were aseptically excised in a laminar flow hood. The tissue was stabilized by gently pressing one end with the left index finger while a sharp blade (moistened with distilled water between cuts) was used to generate longitudinal or transverse sections. Sections were immediately transferred to distilled water to prevent dehydration, mounted on glass slides with coverslips, and visualized under the microscope.

### 4.7. Total RNA Extraction, cDNA Synthesis, and Quantitative PCR (qPCR)

Total RNA was extracted from plant leaves using the FastPure Universal Plant Total RNA Isolation Kit (RC411-01, Vazyme, Nanjing China). cDNA was synthesized from 1 µg of total RNA with the HiScript III Reverse Transcriptase Kit (Vazyme) following the manufacturer’s protocol. Initial PCR screening was performed using Green Taq Mix (P131-01, Vazyme). For quantitative analysis of *GmPDS*, *GmRpp6907*, and *GmRPT4* gene expression across different treatment groups (healthy, TRV:empty, TRV:GmPDS, TRV:Rpp6907, and TRV:RPT4 plants), qPCR was conducted using SYBR Green Master Mix (Bio-Rad, Hercules, CA, USA) on a CFX Connect Real-Time System (Bio-Rad). The thermal cycling conditions consisted of 45 cycles of 95 °C for 10 s (denaturation) and 60 °C for 30 s (annealing/extension), with fluorescence acquisition at each cycle’s end. Soybean β-actin served as the internal reference gene, and relative expression levels were calculated using the 2^−ΔΔCt^ method. *P. pachyrhizi* colonization was quantified by qPCR and normalized to the soybean ubiquitin gene. Three biological replicates were analyzed per tissue type, with each replicate pool consisting of samples from at least five individual plants. All qPCR primers (Table 2) exhibited amplification efficiencies between 90 and 110%, confirming their suitability for quantitative analyses.

### 4.8. P. pachyrhizi Infection and Phenotypic Analyses

A urediniospore suspension was adjusted to 10^5^ spores/mL using 0.01% (*v*/*v*) Tween 20 as a surfactant. Plants were uniformly sprayed with the suspension using a handheld atomizer. Post-inoculation, plants were maintained in a dew chamber at 24 °C with 100% relative humidity for 12 h to facilitate infection, then transferred to greenhouse conditions (20–26 °C, 60% RH, 12-h photoperiod) for 14 days until symptom development. Disease response was categorized as follows: Resistant (R)—all inoculated leaves showing immune (IM) lesions; Incompletely Resistant (IR)—mixed reaction with both reddish-brown (RB) and IM lesions; Susceptible (S)—presence of tan (TAN) lesions. *P. pachyrhizi* colonization was additionally quantified via qPCR to validate visual assessments.

### 4.9. Statistical Analysis

The VIGS experiment set-up included three independent biological replicates. The phenotypic evaluation of gene silencing was calculated with the following formula: gene silencing efficiency (%) = (plants with a 1.5-fold decrease in relative gene expression levels/total number of tested plants) × 100%. The data were analyzed using Excel 2016 software (Microsoft Corp., Redmond, WA, USA), and all statistical analyses were performed with GraphPad Prism (v.9.5). All Student’s *t*-test was used to assess differences between different treatments (* *p* < 0.05, ** *p* < 0.01, *** *p* < 0.001). Values are represented as means ± SD of three independent experiments.

## 5. Conclusions

VIGS has emerged as a powerful reverse genetics tool, particularly valuable for plant species lacking efficient transformation systems. The technology’s advantages stem from its simplicity, robustness, and independence from both stable transformation requirements and complete gene sequence information.

In this study, we have optimized a TRV-based virus-induced gene silencing protocol using an *Agrobacterium*-mediated cotyledonary node for soybean. This approach has effectively silenced the endogenous gene *GmPDS*, the rust-resistant gene *GmRpp6907*, and the defense-related gene *GmRPT4* in soybean. Traditional methods for identifying such genes via soybean transformation technology require approximately 6–7 months. In contrast, our VIGS system can identify candidate genes within 20–30 days, substantially reducing the screening time and accelerating the identification process.

This optimized VIGS protocol not only serves as an efficient tool for functional genomics studies in soybean, particularly for disease resistance gene characterization, but also demonstrates potential for broader applications in resistance research across other crop species. The accelerated gene validation capability provided by this system could significantly contribute to the development of improved soybean cultivars.

## Figures and Tables

**Table 1:** Primers used in vector construction.

Name	Sequence (5′→3′)	Purpose
PDS-F	taaggttaccGAATTCTCTCCGCGTCCTCTAAAAC	Vector construction
PDS-R	atgcccgggcCTCGAGTCCAGGCTTATTTGGCATAGC	
Rpp6907-F	taaggttaccGAATTCTCGGCAAAGTTGGTTTTCATCT	
Rpp6907-R	atgcccgggcCTCGAGCCATTCCTGGGCTCCACATT	
RPT4-F	taaggttaccGAATTCTTTTCCGCACTGATGGTATT	
RPT4-R	atgcccgggcCTCGAGAGAGCAGCCTCGTTCAAGTA	

**Table 2:** Primers used in qPCR.

Name	Sequence (5′→3′)	Amplification Efficiency (%)
Actin-F	ATTGGACTCTGGTGATGGTG	104.2
Actin-R	TCAGCAGAGGTGGTGAACAT	
qPDS-F	TCGCTTCTTCAGACGCCAC	94.5
qPDS-R	TATGCCCAGCATCAGCCAAA	
qRpp6907-F	TCGGCAAAGTTGGTTTTCATCT	93.0
qRpp6907-R	CCATTCCTGGGCTCCACATT	
qRPT4-F	TTTTCCGCACTGATGGTATT	94.9
qRPT4-R	AGCAGCCTCGTTCAAGTA	
RT-Pp-α-tubulin-F	CCAAGGCTTCTTCGTGTTTCA	108.4
RT-Pp-α-tubulin-R	CAAGAGAAGAGCGCCAAACC	
RT-GmUbiquitin-3-F	GTGTAATGTTGGATGTGTTCCC	108.3
RT-GmUbiquitin-3-R	ACACAATTGAGTTCAACACAAACCG	

## Data Availability

The datasets used and analyzed during the current study are available from the corresponding authors upon reasonable request due to privacy.

## Supplementary Materials

The following supporting information can be downloaded at https://www.mdpi.com/article/10.3390/plants14101547/s1, Table S1: Silence efficiency of *GmPDS* in Tianlong 1.
