# Supplementary material for: Efficient Virus-Induced Gene Silencing (VIGS) Method for Discovery of Resistance Genes in Soybean
Source: Plants (Basel). 2025 May 21;14(10):1547. doi: 10.3390/plants14101547 (PMC12115190; doi:10.3390/plants14101547)
Supplement: Supplementary file 1 [file plants-14-01547-s001.zip › plants-3610641-supplementary.pdf]

Table S1. Silence efficiency of *GmPDS* in Tianlong 1

Genotype	No of transformed plants	% of *PDS* transformed plants (photo-bleach)	% of *PDS* negative plants (not photo-bleached)
**Tianlong 1**	**30**	**66.7**	**33.3**
		**73.3**	**26.7**
		**56.7**	**43.3**
		**66.7**	**33.3**
		**53.3**	**46.7**

This data is an independent experiment with 30 plants each time.
